# ABA-Insensitive 5 (ABI5) Is Involved in ABA-Induced Dormancy via Activating *PavCIG1/2* Expression in Sweet Cherries

**DOI:** 10.3390/genes16050596

**Published:** 2025-05-18

**Authors:** Jiyuan Wang, Li Wang, Muhammad Usman, Jie Zhu, Songtao Jiu, Ruie Liu, Caixi Zhang

**Affiliations:** 1 School of Life Sciences, Huaibei Normal University, Huaibei 235000, China; wangjy@chnu.edu.cn (J.W.); zhujie@chnu.edu.cn (J.Z.); 2 Department of Plant Science, School of Agriculture and Biology, Shanghai Jiao Tong University, Minhang, Shanghai 200240, China; wang-li2020@sjtu.edu.cn (L.W.); muhammadusman44@sjtu.edu.cn (M.U.); jiusongtao@sjtu.edu.cn (S.J.); reliu@sjtu.edu.cn (R.L.)

**Keywords:** ABI5, cold tolerance, bud dormancy, ABA, low temperature, *Prunus avium*

## Abstract

Background/Objectives: In perennial plants, developing floral buds survive winter through entering a dormant state, which is induced by low temperature and abscisic acid (ABA). ABA performs vital functions in the dormancy process. ABA-insensitive 5 (ABI5) transcription factor is a key regulator in the ABA signaling pathway. However, little is known about the regulation of ABI5 in the winter dormancy of sweet cherries. Methods: We identified the sweet cherry *ABI5* gene and its expression changes using gene cloning and qRT-PCR. Additionally, we validated the interaction between PavABI5 and PavCIG1/2 using Yeast One-Hybrid and Dual-Luciferase Assays. Results: In this study, we identified a basic leucine zipper (bZIP) family gene *ABI5* from the sweet cherry, which was closely related to *PduABI5* from *Prunus dulcis*, *PpABI5* from *Prunus persica*, *PmABI5* from *Prunus mume*, and *ParABI5* from *Prunus armeniaca*, through phylogenetic tree analysis. The seasonal expression pattern showed that the PavABI5 level was increased during the winter dormancy stage and induced by exogenous ABA. Specifically, we found that the expression of cherry cold-induced genes (*PavCIG1/2*) was positively correlated with *PavABI5* expression. Furthermore, PavABI5 directly bound to the ABRE elements in the *PavCIG1/2* promoters to activate their expression. We further confirmed that the dormancy-associated MADS-box (*DAM*) genes *DAM4* and *DAM5* function downstream of the ABA signaling pathway to regulate bud dormancy in sweet cherries. Conclusions: Our findings suggest a putative regulatory model of ABA-mediated bud-dormancy with *PavABI5*.

## 1. Introduction

Dormancy is an adaptive strategy in perennial trees that enables them to survive winter conditions and resume growth and bloom under favorable environmental conditions. For fruit trees, it is vital to ensure the normal development of floral buds during cold winters. Consequently, plants enter a dormant state before winter to avoid freezing injury [1]. Furthermore, floral buds can also be affected by spring frosts, which will hamper their development and reduce fruit yield [2,3,4].

The dormancy process consists of three distinct physiological stages: paradormancy, endodormancy, and ecodormancy. Among these, endodormancy is crucial for the accumulation of chilling during dormancy. Once sufficient chilling has been accumulated, endodormancy is terminated, and the plant enters ecodormancy. The primary factors inducing endodormancy of perennial plants are low temperature and abscisic acid (ABA) [5,6,7,8]. Sufficient chilling accumulation is essential for dormancy release and flowering [9,10]. In response to low temperatures, C-repeat binding factor (CBF/DREB) transcription factors regulate cold accumulation and dormancy in fruit trees [11,12]. CBFs induce plant dormancy and enhance cold hardiness via affecting the expressions of dormancy-associated MADS-box (DAM) and other cold-regulated (*COR*) genes, including the *COR*, low-temperature induced (*LTI*), responsive to desiccation (*RD*), and early dehydration-inducible (*ERD*) genes [13,14,15]. Additionally, *CBFs* are involved in retarding plant growth and flowering [16,17]. For example, *CBF* homologous genes *PavCIGs*, are involved in flower bud dormancy and flowering in sweet cherry [18]. *DAMs*, CBF target genes, regulate the dormancy processes and chilling requirement [19,20]. The expressions of *DAM1*, *DAM2*, and *DAM4* peak at the paradormancy stage, which is closely associated with growth cessation and bud set in peaches (Prunus persica). The expressions of *DAM5* and *DAM6* peak at the endodormancy stage and decrease toward bud break [21].

ABA functions along with low temperature during the endodormancy phase in some plant species, including apple (*Malus domestica*), peach (*Prunus persica*), and pear (*Pyrus communis*). ABA suppresses bud break, affects flowering time, and enhances plant cold tolerance [22,23,24,25]. The *9-cis-epoxycarotenoid dioxygenase* (*NCED*) genes, which encode a key enzyme in abscisic acid biosynthesis, increase ABA content during the dormancy induction and maintenance stages. In contrast, upregulated *CYP707A* genes promote ABA catabolism when dormancy releases [8,10,26]. In the ABA signaling pathway, an important transcription factor ABA-insensitive 5 (ABI5), belonging to the basic leucine zipper (bZIP) family, is involved in seed germination, drought, and cold tolerance [27,28,29,30]. Allelic variation in *TaABI5-A4* significantly affects seed dormancy in bread wheat [31]. ABI5 regulates downstream genes by binding ABRE elements in their promoters, and most of these downstream genes are stress-related [32,33,34]. Moreover, ABA increases the expression of the *CBF/DREB1* transcription factors in grape buds under low-temperature conditions [35]. Although in pears, which also belong to the Rosaceae family, ABI5-like protein ABF3 has been demonstrated to activate *CBF* and *DAM* [36], the relationship between ABI5 and the *CBF* homologous genes *CIGs* remains unclear in sweet cherries.

Sweet cherries are an important economic crop throughout the world. China has become the largest consumption market for sweet cherries. At present, the total cultivation area of sweet cherries in China is about 3 million mu (approximately 200,000 hectares), with a yield of around 700,000 tons. In some warm temperate regions, the fulfillment of chilling requirements and the breaking of dormancy have become the keys indicating whether sweet cherries can flower and fruit normally. In this study, we identified an ABA signaling pathway gene, *ABI5*, from sweet cherries, which was closely related to PduABI5 from *P. dulcis* and PpABI5 from *P. persica*. The seasonal expression level of *PavABI5* was higher during the winter dormancy stage and was induced by exogenous ABA. Moreover, ABA enhanced the expression of *PavCIG1/2* in combination with low temperature, and PavABI5 directly bound to the ABRE elements of *PavCIG1/2* to increase their transcriptional activity. In addition, the expression of *PavCIG1/2* downstream genes, *PavDAMs*, was also affected by ABA. Our results clarify the potential regulatory mechanisms of ABA-mediated dormancy and cold tolerance in tree fruit species, which will be beneficial to the expansion of sweet cherry cultivation areas in warm temperate regions.

## 2. Materials and Methods

### 2.1. Plant Materials

The 8-year-old sweet cherry (*P. avium* L.) cultivar Royal Lee, with a 400 h chilling requirement [20], was used for this study. The trees were cultivated at the experimental field of Shanghai Jiao Tong University (31.25° N, 121.48° E). The rootstock of Royal Lee was Gisela 6. A pool of floral buds was collected from Royal Lee on 15 July, 15 August, 15 September, 15 October, 15 November, 15 December, 30 December 2020, and 15 January, 1 February, 15 February, 25 February, 5 March, and 15 October 2021. In a previous study, we described the dormancy phases of floral buds, as follows: paradormancy stage—before 1 November; endodormancy stage—1 November to 30 December; ecodormancy stage—1 January to 5 February; budbreak stage—after 5 February [20]. Three individual trees were employed as biological replicates. These buds were frozen in liquid nitrogen and stored at −80 °C.

### 2.2. RNA Isolation and Gene Expression Analysis

Total RNA was isolated from collected samples of Royal Lee using the RNAprep Pure Plant Kit (Tiangen, Beijing, China). The synthesis of first-strand cDNA was carried out with the PrimeScript^TM^ RT reagent Kit (Takara, Shiga, Japan). Gene expression analysis used the CFX96 real-time PCR system (Bio-Rad, Hercules, CA, USA) with TB Green^TM^ Premix Ex Tap^TM^ II (Takara, Shiga, Japan). The 2^−ΔΔCT^ method was employed for data analysis [37]. The primers (Supplementary Table S1) for cloning and qRT-PCR were generated via Primer 5 software, with reference to the sweet cherry genome database in NCBI. GenBank accession numbers of the *PavABI5*, *PavCIG1*, and *PavCIG2* genes were XM_021954506.1, XM_021947969.1, and KC543498.1, respectively. A total of 40 PCR cycles were performed, according to the following temperature program: 95 °C for 30 s, followed by 95 °C for 5 s and 60 °C for 30 s. The phylogenetic trees were achieved using MEGA 6 software via a neighbor-joining method. Homologs of PavABI5 from different species were obtained from NCBI using the BLAST (2.6.0) program.

### 2.3. Yeast One-Hybrid Assays

*PavABI5* coding sequence was inserted into the pB42AD vector, creating a recombinant pB42AD-PavABI5 plasmid. The promoter fragments of *PavCIG1/2* were ligated to the pLacZ vector. These constructs, pB42AD-PavABI5 and pLacZ-PavCIG1, as well as pB42AD-PavABI5 and pLacZ-PavCIG2, were co-transformed into EGY48 cells. Transformed colonies were grown on SD/-Leu/-Ura medium and detected on SD/-Leu/-Ura medium supplemented with X-gal.

### 2.4. Dual-Luciferase Assays

The *PavABI5* coding region was cloned into the pRI101 vector to serve as the effector. The promoter fragments of *PavCIG1/2* were ligated to the pGreen-LUC vector to generate the reporter, and were introduced into GV3101 strains (harboring the pSoup vector). The effector-reporter system was introduced into tobacco leaves via *Agrobacterium tumefaciens* GV3101-mediated transformation. After 72 hours post-infiltration, protein interactions were analyzed through dual-luciferase assays (Vazyme, Nanjing, China).

### 2.5. Subcellular Localization of PavABI5

The *PavABI5* open reading frame (stop codon removed) was amplified with specific primers (Supplementary Table S1), and cloning into the PHB-GFP vector. After transferring both the fusion construct and empty vector into *A. tumefaciens* GV3101, we performed *Agrobacterium* infiltration on tobacco plants at the 5-week growth stage. The GFP fluorescence was detected using a confocal laser scanning microscope after 48–72 h.

### 2.6. ABA and Low Temperature Treatments

For the bud-burst experiment, the 30 cm Royal Lee floral shoots (15 January, ecodormancy stage) were treated with 200 μM ABA, placed in 2 L beakers, for 2 days, and water instead of the ABA solution was used as the control. Two days later, we placed all shoots in 2 L beakers filled with water. Thereafter, images were captured and bud-burst rates were recorded at 0, 9, 14, and 21 days. The growth conditions consisted of a 16/8-hour light/dark cycle at 25/21°C respectively, with 75% humidity and illumination at 300 μmol⋅m^−2^·s^−1^.

For the expression experiment, 200 μM ABA was used for treating the Royal Lee shoots in 2 L beakers, and water instead of the ABA solution was used as the control. Floral buds from the shoots were collected at 24, 48, and 72 h. The growth conditions were maintained at either 25 °C or 10 °C during the photoperiod, with 75% humidity and illumination at 300 μmol⋅m^−2^·s^−1^.

### 2.7. Protein–Protein Interaction Prediction and Expression Pattern Analysis

STRING database analysis [38] was employed to predict PavABI5-interacting proteins, while the transcriptomic data were obtained from NCBI Gene Expression Omnibus (GSE130426) [39]. The TBtools (2.083) program was used to analyze the RNA-seq data and generate heatmaps based on TPM values [40].

## 3. Results

### 3.1. Identification of Sweet Cherry PavABI5 Gene

**Figure 1:**
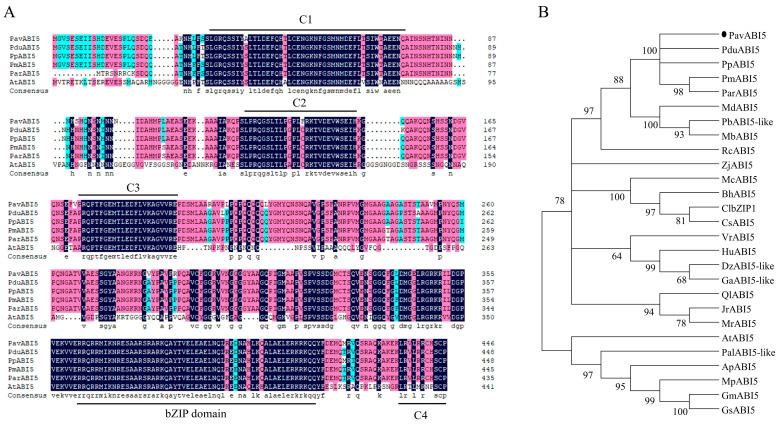
Sequence and phylogenetic analysis of PavABI5. (**A**) Phylogenetic tree based on full-length amino acid sequence for PavABI5 proteins from sweet cherry and other plant species. The conserved domains C1–4 and bZIP are marked with black lines. Dark blue, red, and light blue backgrounds indicate 100%, 75%, and 50% identity, respectively. (**B**) Phylogenetic relationships among ABI5 homologues. The following ABI5 proteins are listed: PduABI5 (XP_034226254.1, *Prunus dulcis*), PpABI5 (XP_007203311.1, *Prunus persica*), PmABI5 (XP_008241247.2, *Prunus mume*), ParABI5 (ADL62859.1, *Prunus armeniaca*), MdABI5 (XP_028946642.1, *Malus domestica*), PbABI5-like (XP_018505200.1, *Pyrus x bretschneideri*), MbABI5 (TQD83332.1, *Malus baccata*), RcABI5 (XP_024186507.1, *Rosa chinensis*), ZjABI5 (XP_015898747.1, *Ziziphus jujuba*), McABI5 (XP_022144787.1, *Momordica charantia*), BhABI5 (XP_038904029.1, *Benincasa hispida*), ClbZIP1 (AOZ56990.1, *Citrullus lanatus*), CsABI5 (XP_004149224.2, *Cucumis sativus*), VrABI5 (XP_034693304.1, *Vitis riparia*), HuABI5 (XP_021290003.1, *Herrania umbratica*), DzABI5-like (XP_022743528.1, *Durio zibethinus*), GaABI5-like (KAA3465066.1, *Gossypium australe*), QlABI5 (XP_030925409.1, *Quercus lobata*), JrABI5 (XP_018813662.1, *Juglans regia*), MrABI5 (KAB1222563.1, *Morella rubra*), AtABI5 (AT2G36270, *Arabidopsis thaliana*), PalABI5-like (XP_028784915.1, *Prosopis alba*), ApABI5 (XP_027363242.1, *Abrus precatorius*), MpABI5 (RDY07453.1, *Mucuna pruriens*), GmABI5 (XP_014618516.1, *Glycine max*), and GsABI5 (XP_014618516.1, *Glycine max*).

An important gene, *PavABI5*, which is involved in the ABA signaling pathway, was investigated in this study. The full-length cDNA sequence of *PavABI5* was cloned from floral bud tissue, revealing a 1344-bp open reading frame encoding a protein of 447 amino acids. The amino acid sequence of the bZIP domain was extremely conserved across six plant species. Three highly conserved regions (C1, C2, and C3) were located at the N-terminus of ABI5 proteins, while a C4 region was found at their C-terminal end. As a result, PavABI5 was identified as belonging to the basic leucine zipper (bZIP) transcription factor family (Figure 1A). To investigate the phylogenetic relationships of PavABI5 with ABI5 proteins from other species, a phylogenetic tree was constructed. The analysis revealed that PavABI5 was closely related to PduABI5 from almond, PpABI5 from peach, PmABI5 from Japanese apricot, and ParABI5 from apricot. These results suggested that PavABI5 might exhibit a similar function to that of ABI5 proteins across these species (Figure 1B).

### 3.2. PavCIG1 and PavCIG2 Are Downstream of PavABI5

**Figure 2:**
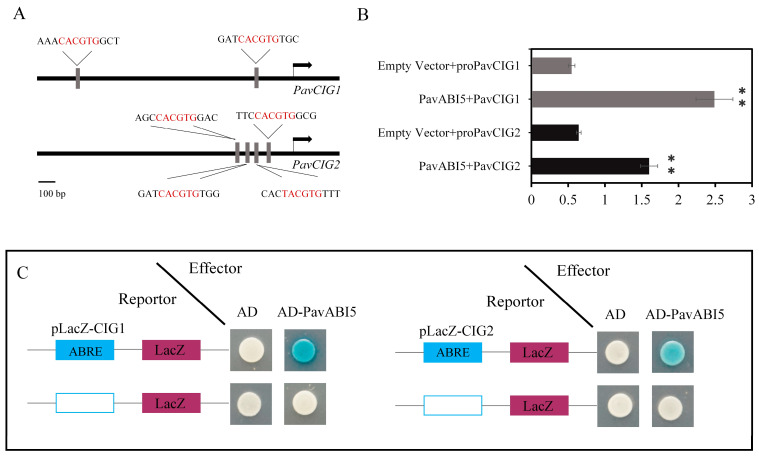
Interactions between PavABI5 and PavCIG1/2. (**A**) Diagram of the *PavCIG1/2* promoters. (**B**) Dual-luciferase assays for examining the interactions between *PavABI5* and *PavCIG1/2* promoters. Empty pGreen-LUC vector was used as a control. As measured by a Student’s *t*-test, an asterisk represents a significant difference of *p* < 0.01. (**C**) Yeast one-hybrid assays for examining the interactions between *PavABI5* and the *PavCIG1/2* promoters. Empty pLacZ vector was used as the control.

We identified two abscisic acid response elements (ABREs) in the *PavCIG1* promoter region (−2000 bp to ATG) and four ABREs in the *PavCIG2* promoter region (−2000 bp to ATG) (Figure 2A). A dual-luciferase assay was conducted to investigate the relationship between PavABI5 and the *PavCIG1/2* promoters. The results revealed that PavABI5 enhanced the activity of both the *PavCIG1* and *PavCIG2* promoters in order to activate their expressions (Figure 2B). Moreover, a Y1H assay was conducted to further confirm the interaction between PavABI5 and the *PavCIG1/2* promoters. The results also indicated that PavABI5 could bind to the promoters of *PavCIG1/2* (Figure 2C).

### 3.3. ABA Delayed Floral Budburst in Sweet Cherry

**Figure 3:**
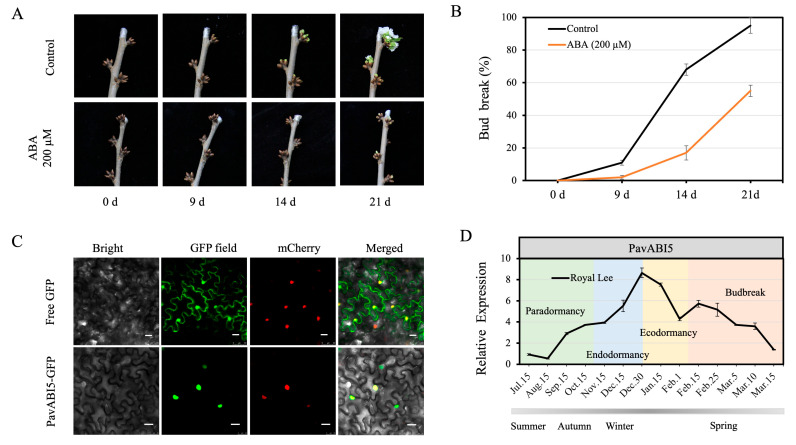
ABA delayed floral bud-burst in sweet cherry. (**A**) Sweet cherry shoots were placed in solution (200 μM ABA or water) in 2 L beakers for 2 d; thereafter, images were recorded at 0, 9, 14, 21 d. The growth conditions were maintained at 25 °C for a 16 h light and 21 °C for an 8 h dark photoperiod, with 75% humidity and 300 μmol⋅m^−2^·s^−1^ light intensity. (**B**) Bud-burst rate was recorded at 0, 9, 14, 21 d after ABA treatment. (**C**) Subcellular localization of PavABI5. The 35S:PavABI5-GFP or 35S:GFP vectors were introduced into *A. tumefaciens* GV3101. The red fluorescent protein containing NLS-mCherry was co-expressed. Bars, 25 μm. (**D**) Seasonal expression of *PavABI5* was evaluated in floral buds of Royal Lee. The error bars represent the standard deviations (SDs) of three biological replicates.

The sweet cherry flower buds collected from shoots on 15 January (side green stage) were treated with 200 μM ABA. After 14 days, the control shoots exhibited a bud-burst rate of approximately 68%. However, these flower buds treated with ABA remained in a dormant state. After 21 days, the control flower buds began to flower, with a bud-burst rate of approximately 95%, while the ABA-treated flower buds broke dormancy with a bud-burst rate of approximately 53% (Figure 3A,B).

The coding regions of *PavABI5* cDNA, excluding its stop codon, were inserted into the PHB-GFP vector to produce the fusion construct PavABI5-GFP. The fusion plasmids and the control vector (PHB-GFP) were introduced into *A. tumefaciens* GV3101. Then, they were infiltrated into the leaves of 5-week-old tobacco plants. After 48–72 h of incubation, the GFP fluorescence revealed that PavABI5 was expressed in the cell nucleus compared with the results for NLS-mCherry (Figure 3C).

To explore the expression pattern of *PavABI5* across different seasons in sweet cherry, real-time quantitative PCR was performed on floral buds. As shown in Figure 3D, *PavABI5* transcription was barely detectable in summer. *PavABI5* expression began to accumulate from autumn, and reached a peak on 30 December during winter. In addition, our previous research suggested that *PavCIG1* and *PavCIG2* exhibited similar expression patterns to those of *PavABI5* [18].

### 3.4. Gene Expression Analysis Through ABA and Low Temperature Treatments

**Figure 4:**
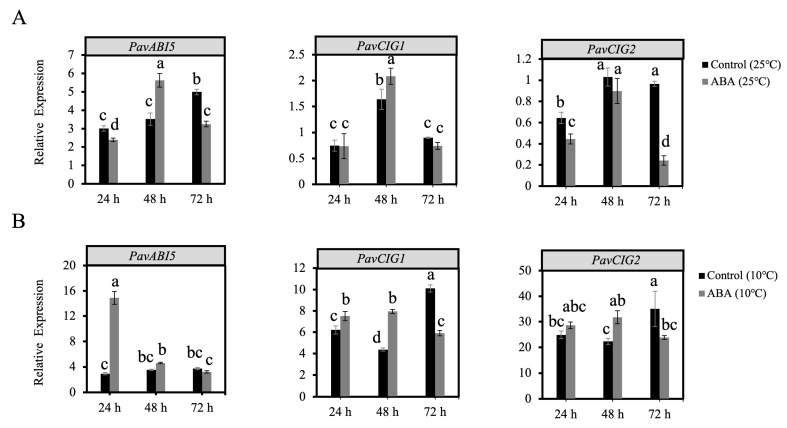
Expression patterns of *PavABI5* and *PavCIG1/2* after ABA and low temperature treatments. (**A**) After ABA treatment, floral buds were collected at 24, 48, and 72 h under a temperature of 25 °C. The expressions of *PavABI5* and *PavCIGs* were detected by qRT-PCR assay. (**B**) After ABA treatment, floral buds collected at 24, 48, and 72 h under a temperature of 10 °C were used to detect the expressions of *PavABI5* and *PavCIGs*. Error bars represent the standard deviation of three biological replicates. Different letters indicate significant differences (*p* < 0.01).

To assess the effect of ABA and low temperature on the downstream genes, *ABI5* and CIGs, we examined their expression patterns in the flower buds. As shown in Figure 4A, after a 48 h ABA treatment, the expression of *PavABI5* was increased significantly at 25 °C. Moreover, the expression of *PavABI5* was increased rapidly under the low temperature of 10 °C, and reached a higher level than that of the control (10 °C) during the first 24 h (Figure 4B). *PavCIG1* and *PavCIG2* were expressed at higher levels after ABA treatment (10 °C) than in the control (10 °C) at 24 and 48 h (Figure 4B).

### 3.5. Expression Analysis of Downstream Gene DAMs After ABA and Cold Treatments

**Figure 5:**
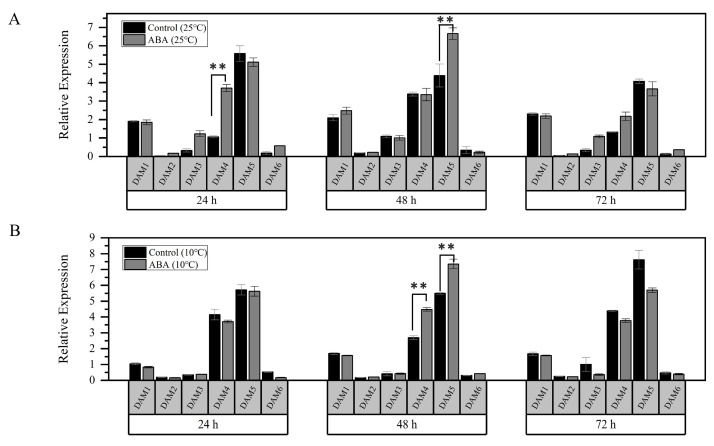
Expression patterns of *PavCIG1/2* downstream of gene *PavDAM1-6* after ABA and low temperature treatments. (**A**) After ABA treatment, floral buds were collected at 24, 48, and 72 h under a temperature of 25 °C. The expressions of *PavDAM1-6* were detected by qRT-PCR assay. (**B**) After ABA treatment, floral buds collected at 24, 48, and 72 h under a temperature of 10 °C were used to detect the expressions of *PavDAM1-6*. Error bars represent the standard deviation of three biological replicates. (** *p* ≤ 0.01, Student’s *t*-test).

*DAMs* are selectively affected by *CBFs* in the regulation of the bud dormancy process [15,19]. In this study, we found that the expression levels of the major genes *PavDAM4* and *PavDAM5* were significantly increased after ABA treatment (25 °C) at 24 h and 48 h, respectively (Figure 5A). Moreover, the expressions of both genes were significantly higher than those of the control after a 48 h ABA treatment under low temperature conditions (10 °C) (Figure 5B). However, no direct interactions were observed between PavCIG1/2 and PavDAM4/5 (Supplementary Figure S1). These results suggested that *PavDAM4* and *PavDAM5* may act downstream of ABA signaling to modulate dormancy.

### 3.6. Prediction of Protein–Protein Interactions Between PavABI5 and Other Proteins

**Figure 6:**
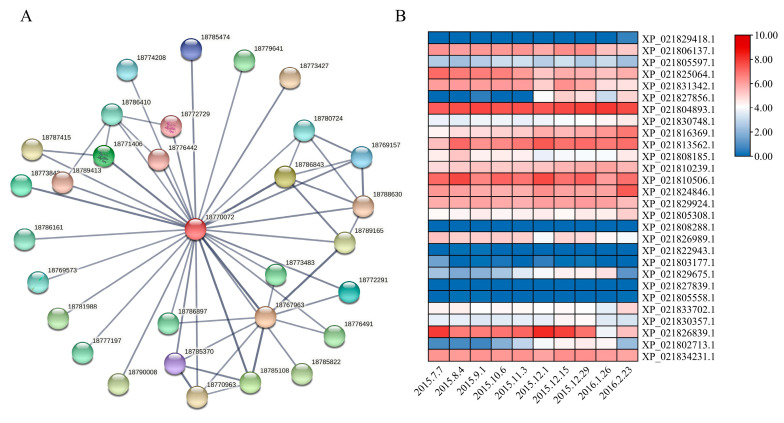
The interaction prediction between PavABI5 and other proteins. (**A**) Prediction model diagram of the interaction between PavABI5 and other proteins through the STRING database. PavABI5 homologous protein PpABI5 (18770072, XP_020423436, *Prunus persica*) was used to predict the interaction proteins. (**B**) Seasonal expression patterns of predicted genes from 7 June 2015 to 23 February 2016 in the sweet cherry floral buds. Expression values for these genes were transformed by log_2_ (TPM+1). Red and white cells indicate relative higher or lower expression.

To further explore the function of PavABI5, we constructed a protein interaction network using the STRING database (Figure 6A) [38]. As shown in Table 1, nearly 30 predicted proteins (Score > 0.7) were likely to interact with PavABI5. These proteins included ABI3/4, E3 ubiquitin-protein ligase KEG, serine/threonine-protein kinase SRK2I, E3 SUMO-protein ligase SIZ1, flowering time control protein FCA, bZIP transcription factor TGA10, and others. Then, we analyzed the expression patterns of these 30 genes using transcriptomic data from sweet cherry floral buds collected between July 2015 and February 2016 (Figure 6B) [39]. The results revealed that some genes, such as XP_021829418.1 (*ABI3*), XP_021808288.1 (*FCA*), and XP_021822943.1 (*FUS3*), were expressed at low levels during this period, while others, such as XP_021804893.1 (*4A-2-like*), XP_021813562.1 (*SAPK3*), and XP_021834231.1 (*Cul4*), were consistently expressed at high levels. Notably, similar expression patterns to that of *PavABI5* were observed, including the TGA family genes *TGA9* (XP_021829675.1), *TGA10* (XP_021802713.1), and *SLE1-like* (XP_021827856.1). Additionally, the expression of E3 SUMO-protein ligase *SIZ1* (XP_021831342.1) significantly increased during the winter period.

## 4. Discussion

ABA is a stress hormone and a central regulator of dormancy. Genes involved in ABA synthesis and catabolism play important roles in dormancy. In *Arabidopsis*, three *NCED*-related genes involved in ABA biosynthesis, *NCED5*, *NCED6*, and *NCED9*, have been proven to regulate seed development and dormancy [41,42]. For ABA catabolism, the expression of *CYP707A* genes reduces the concentration of ABA, promoting seed dormancy release during the maturation stage [43]. Low temperature can enhance dormancy by increasing ABA content and decreasing *CYP707A* expression in *Arabidopsis* seeds [44]. Similarly, ABA plays a key role in regulating bud dormancy [45,46]. It induces shoot growth cessation and initiates dormancy in autumn, with peak ABA concentrations observed in mid-winter, followed by a decline in December in apple buds [47]. In grape buds, ABA content begins to increase during para- and endodormancy, then decreases towards dormancy release [48]. The breaking of pear flower buds is inhibited after ABA applications, which affects the endodormancy induction [49]. In our research, the bud-break percentage of sweet cherries was decreased after ABA treatment compared to that of the control (Figure 3A). Recently, ABA content has been proposed as a determining factor for assessing dormancy status in sweet cherries [25,50], and genes involved in ABA-related pathways were central in the transcriptomic analysis of flower bud dormancy [39].

ABI5, a core component of the ABA signaling pathway, controls the dormancy process. The ABA receptors PYR/PYL/RCAR positively regulate seed dormancy by inhibiting the PP2C protein [51], while PP2Cs negatively regulate ABA signaling by interacting with SnRK2s and dephosphorylating them [52]. Downstream of SnRK2s, *ABI5*, participates in ABA-mediated seed dormancy [53,54]. Similar to other species, PavABI5 belongs to the bZIP family and contains conserved domains C1-4 (Figure 1) [55,56,57]. In gladiolus, *GhABI5* is expressed in dormant organs (corm, cormel, stolon, stamen) and induced by drought and ABA [58]. In our results, the expression of *PavABI5* reached a peak in mid-winter, and was strongly induced by exogenous ABA (Figure 3D and Figure 4A). The expression of *PavABI5* is also affected by the sumoylation of SUMO E3 ligase SIZ1, an interaction protein (Figure 6) [59]. It is predicted that PavABI5 interacts with PavABI3 in the sweet cherries to regulate the bud-break. Meanwhile, *ABI5* acts as a downstream gene of ABI3 in *Arabidopsis* [60]. The interactions of PavABI5 with PavFCA, PavTGA9, and PavTGA10 may be responsible for the delay in flowering time and flower development in sweet cherries [61,62].

**Figure 7:**
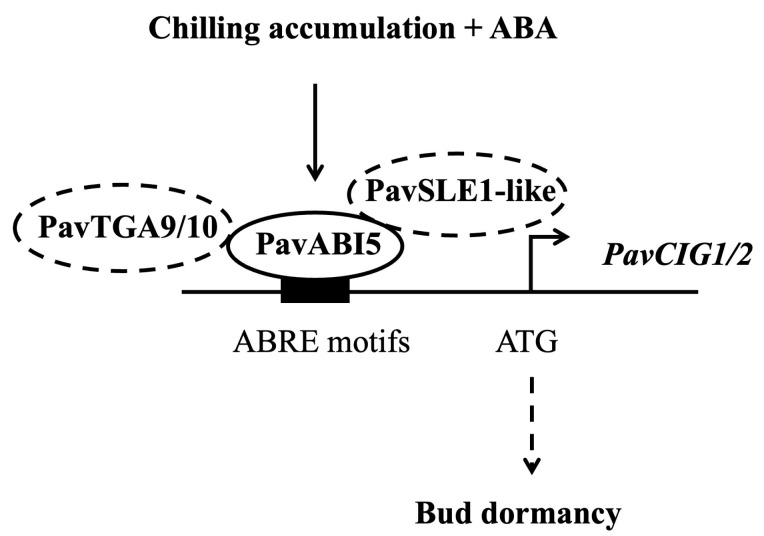
A regulation model of PavABI5 in sweet cherry flower bud dormancy.

Buds of perennial fruit trees typically enter a dormancy state during winter in order to survive the low temperatures. For perennial fruit trees such as pear and apple, dormancy induction is established by low temperature rather than by the photoperiod [5]. In the presence of ABA and low temperature, *PavABI5* was expressed more rapidly and reached higher levels, whereas low temperature alone had minimal effects on its expression, suggesting that low temperature enhanced the expression of *PavABI5* in an ABA-dependent manner (Figure 4). Banana MaABI5 interacts with MaC3HC4-1 to participate in the regulation of cold tolerance [29]. Moreover, ABI5 induces the expressions of ABA-responsive genes by binding to the ABA responsive elements (ABRE; ACGTGG/TC) in their promoter regions [32,59]. We found that the expressions of *PavCIG1* and *PavCIG2* were increased in response to exogenous ABA treatment compared with the results for the control under low temperature conditions (Figure 4B). In grapes, ABA treatment upregulates *CBF1/2/3* transcripts, enhancing the freezing tolerance [63]. Similarly, the expression of cassava *MeCBF1* is highly responsive to cold and ABA treatment [64]. Moreover, there were two ABRE elements in the promoter of the cold-induced gene *PavCIG1* and four ABRE elements in the promoter of *PavCIG2*. Additional experiments proved the interactions between PavABI5 and PavCIG1/2 (Figure 2). In pears, the ABI5 homologous protein PpyABF3 activates the *PpyCBF4* promoter by binding to its ABREs to regulate bud dormancy [36]. Previous research indicates that overexpressing the *CBF* gene results in dwarf plants, delayed bud-break, and increased cold tolerance [13,65,66,67]. Similar to the results for these species, the ectopic expression of sweet cherry *PavCIG1* and *PavCIG2* in *Arabidopsis* also delayed flowering [18]. *DAMs,* CBF downstream genes, are also regulated by the AREB transcription factor and function to inhibit bud growth and enhance bud dormancy [14,36,68,69]. In our results, we found that the expressions of *PavDAM4* and *PavDAM5* were significantly induced in response to exogenous ABA treatment under the temperatures of 25 °C and 10 °C (Figure 5). However, no direct interactions were observed between PavABI5 and PavDAM4/5 (Supplementary Figure S2). As a result, PavABI5 might be involved in regulating bud dormancy and cold tolerance via the CBF-mediated pathway in sweet cherries (Figure 7).

## Figures and Tables

**Table 1:** Predicted proteins interacting with PavABI5.

Gene ID.	Protein ID *Prunus persica*	Peach Protein Name	Score	Protein ID *Prunus avium*
18767963	XP_007200956	B3 domain-containing transcription factor ABI3	1.0	XP_021829418.1
18786843	XP_007220580	E3 ubiquitin-protein ligase KEG	0.99	XP_021806137.1
18785108	XP_007221036	ethylene-responsive transcription factor ABI4	0.97	XP_021805597.1
18771406	XP_007202101	serine/threonine-protein kinase SRK2I	0.92	XP_021825064.1
18773843	XP_020423164	E3 SUMO-protein ligase SIZ1	0.91	XP_021831342.1
18772291	XP_007206180	protein SLE1-like	0.83	XP_021827856.1
18769157	XP_007204373	eukaryotic initiation factor 4A-2-like	0.83	XP_021804893.1
18785370	XP_007218800	xanthoxin dehydrogenase	0.82	XP_021830748.1
18772729	XP_007205499	serine/threonine-protein kinase SAPK1	0.82	XP_021816369.1
18776442	XP_020419823	serine/threonine-protein kinase SAPK3	0.82	XP_021813562.1
18789413	XP_007222500	serine/threonine-protein kinase SAPK2	0.82	XP_021808185.1
18788630	XP_007222234	WD repeat-containing protein DWA2	0.81	XP_021810239.1
18773427	XP_007205151	CBL-interacting serine/threonine-protein kinase 11	0.81	XP_021810506.1
18770963	XP_020423783	zeaxanthin epoxidase	0.79	XP_021824846.1
18787415	XP_020413451	abscisic acid-insenstivie 5-like protein 2	0.78	XP_021829924.1
18789165	XP_007222944	E3 ubiquitin-protein ligase AIP2	0.78	XP_021805308.1
18790008	XP_020419715	flowering time control protein FCA	0.78	XP_021808288.1
18785822	XP_007219735	E3 SUMO-protein ligase SIZ1-like	0.77	XP_021826989.1
18781988	XP_020416273	B3 domain-containing transcription factor FUS3	0.75	XP_021822943.1
18776491	XP_007209687	protein MOTHER of FT and TFL1 isoform X2	0.75	XP_021803177.1
18779641	XP_007213620	transcription factor TGA9	0.74	XP_021829675.1
18773483	XP_007206180	late embryogenesis abundant protein EMB564-like	0.74	XP_021827839.1
18786897	XP_007219705	em protein H5	0.74	XP_021805558.1
18777197	XP_007209879	probable aminotransferase ACS10	0.74	XP_021833702.1
18786161	XP_007219026	probable aminotransferase ACS12	0.74	XP_021830357.1
18786410	XP_007220143	probable protein phosphatase 2C 75	0.72	XP_021826839.1
18769573	XP_007204568	bZIP transcription factor TGA10	0.72	XP_021802713.1
18780724	XP_007214632	cullin-4	0.72	XP_021834231.1

## Data Availability

Data are contained within the article and Supplementary Materials.

## Supplementary Materials

The following supporting information can be downloaded at: https://www.mdpi.com/article/10.3390/genes16050596/s1, Figure S1: Yeast one-hybrid assay was performed to confirm the interactions between PavCIG1/2 and the PavDAM4/5 promoters; Figure S2: Interactions between PavABI5 and PavDAM4/5; Table S1: List of primer sequences used in this study.

## References

[1] Arora R., Rowland L.J., Tanino K. (2003). Induction and release of bud dormancy in woody perennials: A science comes of age. HortScience.

[2] Rodrigo J. (2000). Spring frosts in deciduous fruit trees-morphological damage and flower hardiness. Sci. Hortic..

[3] Salazar-Gutiérrez M.R., Chaves B., Hoogenboom G. (2016). Freezing tolerance of apple flower buds. Sci. Hortic..

[4] Kaya O., Kose C., Gecim T. (2018). An exothermic process involved in the late spring frost injury to flower buds of some apricot cultivars (*Prunus armenica* L.). Sci. Hortic..

[5] Heide O.M., Prestrud A.K. (2005). Low temperature, but not photoperiod, controls growth cessation and dormancy induction and release in apple and pear. Tree Physiol..

[6] Kovaleski A.P. (2024). The potential for an increasing threat of unseasonal temperature cycles to dormant plants. New Phytol..

[7] Yuan Y., Zeng L., Kong D., Mao Y., Xu Y., Wang M., Zhao Y., Jiang C., Zhang Y., Sun D. (2024). Abscisic acid–induced transcription factor PsMYB306 negatively regulates tree peony bud dormancy release. Plant Physiol..

[8] Zheng C., Halaly T., Acheampong A.K., Takebayashi Y., Jikumaru Y., Kamiya Y., Or E. (2015). Abscisic acid (ABA) regulates grape bud dormancy, and dormancy release stimuli may act through modification of ABA metabolism. J. Exp. Bot..

[9] Alburquerque N., García-Montiel F., Carrillo A., Burgos L. (2008). Chilling and heat requirements of sweet cherry cultivars and the relationship between altitude and the probability of satisfying the chill requirements. Environ. Exp. Bot..

[10] Wang L., Zhang L., Ma C., Xu W., Liu Z., Zhang C., Matthew D.W., Wang S. (2016). Impact of chilling accumulation and hydrogen cyanamide on floral organ development of sweet cherry in a warm region. J. Integr. Agr..

[11] Artlip T., McDermaid A., Ma Q., Wisniewski M. (2019). Differential gene expression in non-transgenic and transgenic “M. 26” apple overexpressing a peach *CBF* gene during the transition from eco-dormancy to bud break. Hortic. Res..

[12] Barros P.M. (2012). Insights into the Role of Almond CBF Transcription Factors in the Environmental Control of Cold Acclimation and Dormancy Break. Doctoral Dissertation.

[13] Artlip T.S., Wisniewski M.E., Norelli J.L. (2014). Field evaluation of apple overexpressing a peach *CBF* gene confirms its effect on cold hardiness, dormancy, and growth. Environ. Exp. Bot..

[14] Niu Q., Li J., Cai D., Qian M., Jia H., Bai S., Hussain S., Liu G., Teng Y., Zheng X. (2016). Dormancy-associated MADS-box genes and microRNAs jointly control dormancy transition in pear (*Pyrus pyrifolia* white pear group) flower bud. J. Exp. Bot..

[15] Li J., Yan X., Yang Q., Ma Y., Yang B., Tian J., Teng Y., Bai S. (2019). PpCBFs selectively regulate *PpDAMs* and contribute to the pear bud endodormancy process. Plant Mol. Biol..

[16] Park S., Lee C.M., Doherty C.J., Gilmour S.J., Kim Y., Thomashow M.F. (2015). Regulation of the Arabidopsis CBF regulon by a complex low-temperature regulatory network. Plant J..

[17] Artlip T.S., Wisniewski M.E., Arora R., Norelli J.L. (2016). An apple rootstock overexpressing a peach *CBF* gene alters growth and flowering in the scion but does not impact cold hardiness or dormancy. Hortic. Res..

[18] Wang J., Liu X., Sun W., Xu Y., Sabir I.A., Abdullah M., Wang S., Jiu S., Zhang C. (2021). Cold induced genes (*CIGs*) regulate flower development and dormancy in *Prunus avium* L.. Plant Sci..

[19] Zhao K., Zhou Y., Ahmad S., Yong X., Xie X., Han Y., Li Y., Zhang Q. (2018). *PmCBFs* synthetically affect *PmDAM6* by alternative promoter binding and protein complexes towards the dormancy of bud for *Prunus mume*. Sci. Rep..

[20] Wang J., Gao Z., Li H., Jiu S., Qu Y., Wang L., Ma C., Xu W., Wang S., Zhang C. (2020). Dormancy-associated MADS-Box (*DAM*) genes influence chilling requirement of sweet cherries and co-regulate flower development with *SOC1* gene. Int. J. Mol. Sci..

[21] Li Z., Reighard G.L., Abbott A.G., Bielenberg D.G. (2009). Dormancy-associated MADS genes from the *EVG* locus of peach [*Prunus persica* (L.) Batsch] have distinct seasonal and photoperiodic expression patterns. J. Exp. Bot..

[22] Hu C., Wang M., Zhu C., Wu S., Li J., Yu J., Hu Z. (2024). A transcriptional regulation of ERF15 contributes to ABA-mediated cold tolerance in tomato. Plant Cell Environ..

[23] Ali A., Zareen S., Park J., Khan H.A., Lim C.J., Bader Z.E., Hussain S., Chung W.S., Gechev T., Pardo J.M. (2024). ABA INSENSITIVE 2 promotes flowering by inhibiting OST1/ABI5-dependent FLOWERING LOCUS C transcription in Arabidopsis. J. Exp. Bot..

[24] Wang J., Zhang L., Sun W., Wang L., Liu X., Jiu S., Liu R., Zhang C. (2024). N6-methyladenosine RNA methylation is important for dormancy release in sweet cherry. Sci. Hortic..

[25] Vimont N., Schwarzenberg A., Domijan M., Donkpegan A.S., Beauvieux R., Le D.L., Arkoun M., Jamois F., Yvin J., Wigge A.P. (2021). Fine tuning of hormonal signaling is linked to dormancy status in sweet cherry flower buds. Tree Physiol..

[26] Zheng C., Acheampong A.K., Shi Z., Mugzech A., Halaly-Basha T., Shaya F., Sun Y., Colova V., Mosquna A., Ophir R. (2018). Abscisic acid catabolism enhances dormancy release of grapevine buds. Plant Cell Environ..

[27] Finkelstein R.R., Lynch T.J. (2000). The *Arabidopsis* abscisic acid response gene *ABI5* encodes a basic leucine zipper transcription factor. Plant Cell.

[28] Mittal A., Gampala S.S., Ritchie G.L., Payton P., Burke J.J., Rock C.D. (2014). Related to ABA-Insensitive3 (ABI3)/Viviparous1 and AtABI5 transcription factor coexpression in cotton enhances drought stress adaptation. Plant Biotechnol J..

[29] Chen J., Li Y., Li F., Wu Q., Jiang Y., Yuan D. (2018). Banana MaABI5 is involved in ABA-induced cold tolerance through interaction with a RING E3 ubiquitin ligase; MaC3HC4-1. Sci. Hortic..

[30] Zhao H., Nie K., Zhou H., Yan X., Zhan Q., Zheng Y., Song C.P. (2020). ABI5 modulates seed germination via feedback regulation of the expression of the *PYR/PYL/RCAR ABA* receptor genes. New Phytol..

[31] Han Y., Wang Z., Han B., Zhang Y., Liu J., Yang Y. (2024). Allelic variation of TaABI5-A4 significantly affects seed dormancy in bread wheat. Theor. Appl. Genet..

[32] Carles C., Bies-Etheve N., Aspart L., Léon-Kloosterziel K.M., Koornneef M., Echeverria M., Delseny M. (2002). Regulation of *Arabidopsis thaliana Em* genes: Role of ABI5. Plant J..

[33] Yang X., Yang Y.N., Xue L.J., Zou M.J., Liu J.Y., Chen F., Xue H.W. (2011). Rice ABI5-Like1 regulates abscisic acid and auxin responses by affecting the expression of ABRE-containing genes. Plant Physiol..

[34] Yan F., Deng W., Wang X., Yang C., Li Z. (2012). Maize (*Zea mays* L.) homologue of ABA-insensitive (*ABI*) *5* gene plays a negative regulatory role in abiotic stresses response. Plant Growth Regul..

[35] Rubio S., Noriega X., Pérez F.J. (2019). Abscisic acid (ABA) and low temperatures synergistically increase the expression of CBF/DREB1 transcription factors and cold-hardiness in grapevine dormant buds. Ann. Bot..

[36] Yang Q., Yang B., Li J., Wang Y., Tao R., Yang F., Wu X., Yan X., Ahmad M., Shen J. (2020). ABA-responsive ABRE-BINDING FACTOR3 activates *DAM3* expression to promote bud dormancy in Asian pear. Plant Cell Environ..

[37] Livak K.J., Schmittgen T.D. (2001). Analysis of relative gene expression data using real-time quantitative PCR and the 2^−ΔΔCT^ method. Methods.

[38] Szklarczyk D., Franceschini A., Wyder S., Forslund K., Heller D., Huerta-Cepas J., Simonovic M., Roth A., Santos A., Tsafou K.P. (2015). STRING v10: Protein–protein interaction networks.; integrated over the tree of life. Nucleic Acids Res..

[39] Vimont N., Fouché M., Campoy J.A., Tong M., Arkoun M., Yvin J.C., Wigge P.A., Dirlewanger E., Cortijo S., Wenden B. (2019). From bud formation to flowering: Transcriptomic state defines the cherry developmental phases of sweet cherry bud dormancy. BMC Genom..

[40] Chen C., Chen H., Zhang Y., Thomas H.R., Frank M.H., He Y., Xia R. (2020). TBtools: An integrative toolkit developed for interactive analyses of big biological data. Mol. Plant.

[41] Lefebvre V., North H., Frey A., Sotta B., Seo M., Okamoto M., Nambara E., Marion-Poll A. (2006). Functional analysis of Arabidopsis *NCED6* and *NCED9* genes indicates that ABA synthesized in the endosperm is involved in the induction of seed dormancy. Plant J..

[42] Frey A., Effroy D., Lefebvre V., Seo M., Perreau F., Berger A., Sechet J., To A., North H.M., Marion-Poll A. (2012). Epoxycarotenoid cleavage by NCED5 fine-tunes ABA accumulation and affects seed dormancy and drought tolerance with other NCED family members. Plant J..

[43] Okamoto M., Kuwahara A., Seo M., Kushiro T., Asami T., Hirai N., Kamiya Y., Koshiba T., Nambara E. (2006). CYP707A1 and CYP707A2, which encode abscisic acid 8′-hydroxylases.; are indispensable for proper control of seed dormancy and germination in Arabidopsis. Plant Physiol..

[44] Kendall S.L., Hellwege A., Marriot P., Whalley C., Graham I.A., Penfield S. (2011). Induction of dormancy in *Arabidopsis* summer annuals requires parallel regulation of DOG1 and hormone metabolism by low temperature and CBF transcription factors. Plant Cell.

[45] Cooke J.E., Eriksson M.E., Junttila O. (2012). The dynamic nature of bud dormancy in trees: Environmental control and molecular mechanisms. Plant Cell Environ..

[46] Zhao Y., Gao J., Im K.J., Chen K., Bressan R.A., Zhu J.K. (2017). Control of plant water use by ABA induction of senescence and dormancy: An overlooked lesson from evolution. Plant Cell Physiol..

[47] Guak S., Fuchigami L.H. (2001). Effects of applied ABA on growth cessation, bud dormancy, cold acclimation, leaf senescence and N mobilization in apple nursery plants. J. Hortic. Sci. Biotech.

[48] Or E., Belausov E., Popilevsky I., Bental Y. (2000). Changes in endogenous ABA level in relation to the dormancy cycle in grapevines grown in a hot climate. J. Hortic. Sci. Biotech.

[49] Li J., Xu Y., Niu Q., He L., Teng Y., Bai S. (2018). Abscisic acid (ABA) promotes the induction and maintenance of pear (*Pyrus pyrifolia* white pear group) flower bud endodormancy. Int. J. Mol. Sci..

[50] Chmielewski F.M., Gotz K., Homann T., Huschek G., Rawel H. (2017). Identification of endodormancy release for cherries (*Prunus avium* L.) by abscisic acid and sugars. J. Hortic..

[51] Park S.Y., Fung P., Nishimura N., Jensen D.R., Fujii H., Zhao Y., Lumba S., Santiago J., Rodrigues A., Chow T.-F.F. (2009). Abscisic acid inhibits type 2C protein phosphatases via the PYR/PYL family of START proteins. Science.

[52] Vlad F., Rubio S., Rodrigues A., Sirichandra C., Belin C., Robert N., Leung J., Rodriguez P.L., Laurière C., Merlot S. (2009). Protein phosphatases 2C regulate the activation of the Snf1-related kinase OST1 by abscisic acid in *Arabidopsis*. Plant Cell.

[53] Kong Y., Chen S., Yang Y., An C. (2013). ABA-insensitive (ABI) 4 and ABI5 synergistically regulate *DGAT1* expression in *Arabidopsis* seedlings under stress. FEBS Lett..

[54] Utsugi S., Ashikawa I., Nakamura S., Shibasaka M. (2020). TaABI5, a wheat homolog of Arabidopsis thaliana ABA insensitive 5, controls seed germination. J. Plant Res..

[55] Bensmihen S., Rippa S., Lambert G., Jublot D., Pautot V., Granier F., Giraudat J., Parcy F. (2002). The homologous ABI5 and EEL transcription factors function antagonistically to fine-tune gene expression during late embryogenesis. Plant Cell.

[56] Skubacz A., Daszkowska-Golec A., Szarejko I. (2016). The role and regulation of ABI5 (ABA-Insensitive 5) in plant development.; abiotic stress responses and phytohormone crosstalk. Front. Plant Sci..

[57] Wang Y.H., Que F., Li T., Zhang R.R., Khadr A., Xu Z.S., Tian Y.S., Xiong A.S. (2021). DcABF3, an ABF transcription factor from carrot.; alters stomatal density and reduces ABA sensitivity in transgenic *Arabidopsis*. Plant Sci..

[58] Wu J., Seng S., Sui J., Vonaartis E., Luo X., Gong B., Liu C., Wu C., Liu C., Zhang F. (2015). Gladiolus hybridus ABSCISIC ACID INSENSITIVE 5 (GhABI5) is an important transcription factor in ABA signaling that can enhance Gladiolus corm dormancy and Arabidopsis seed dormancy. Front. Plant Sci..

[59] Miura K., Lee J., Jin J.B., Yoo C.Y., Miura T., Hasegawa P.M. (2009). Sumoylation of ABI5 by the *Arabidopsis* SUMO E3 ligase SIZ1 negatively regulates abscisic acid signaling. Proc. Natl. Acad. Sci. USA.

[60] Lopez-Molina L., Mongrand S., McLachlin D.T., Chait B.T., Chua N.H. (2002). ABI5 acts downstream of ABI3 to execute an ABA-dependent growth arrest during germination. Plant J..

[61] Wang Y., Tao Z., Wang W., Filiault D., Qiu C., Wang C., Wang H., Rehman S., Shi J., Zhang Y. (2020). Molecular variation in a functionally divergent homolog of FCA regulates flowering time in *Arabidopsis thaliana*. Nat. Commun..

[62] Murmu J., Bush M.J., DeLong C., Li S., Xu M., Khan M., Malcolmson C., Fobert P.R., Zachgo S., Hepworth S.R. (2010). Arabidopsis basic leucine-zipper transcription factors TGA9 and TGA10 interact with floral glutaredoxins ROXY1 and ROXY2 and are redundantly required for anther development. Plant Physiol..

[63] Xiao H., Siddiqua M., Braybrook S., Nassuth A. (2006). Three grape *CBF/DREB1* genes respond to low temperature, drought and abscisic acid. Plant Cell Environ..

[64] An D., Ma Q., Wang H., Yang J., Zhou W., Zhang P. (2017). Cassava C-repeat binding factor 1 gene responds to low temperature and enhances cold tolerance when overexpressed in Arabidopsis and cassava. Plant Mol. Biol..

[65] Siddiqua M., Nassuth A. (2011). *Vitis CBF1* and *Vitis CBF4* differ in their effect on *Arabidopsis* abiotic stress tolerance.; development and gene expression. Plant Cell Environ..

[66] Wang Z., Liu J., Guo H., He X., Wu W., Du J., Zhang Z., An X. (2014). Characterization of two highly similar *CBF/DREB1*-like genes, *PhCBF4a* and *PhCBF4b*, in *Populus hopeiensis*. Plant Physiol. Bioch..

[67] Wisniewski M., Norelli J., Artlip T. (2015). Overexpression of a peach *CBF* gene in apple: A model for understanding the integration of growth, dormancy, and cold hardiness in woody plants. Front. Plant Sci..

[68] Tuan P.A., Bai S., Saito T., Ito A., Moriguchi T. (2017). Dormancy-Associated MADS-Box (*DAM*) and the abscisic acid pathway regulate pear endodormancy through a feedback mechanism. Plant Cell Physiol..

[69] Yamane H., Wada M., Honda C., Matsuura T., Ikeda Y., Hirayama T., Osako Y., Gao-Takai M., Kojima M., Sakakibara H. (2019). Overexpression of *Prunus DAM6* inhibits growth.; represses bud break competency of dormant buds and delays bud outgrowth in apple plants. PLoS ONE.

