# Supplementary material for: ABA-Insensitive 5 (ABI5) Is Involved in ABA-Induced Dormancy via Activating PavCIG1/2 Expression in Sweet Cherries
Source: Genes (Basel). 2025 May 18;16(5):596. doi: 10.3390/genes16050596 (PMC12111482; doi:10.3390/genes16050596)
Supplement: Supplementary file 1 [file genes-16-00596-s001.zip › genes-3643477-supplementary.pdf]

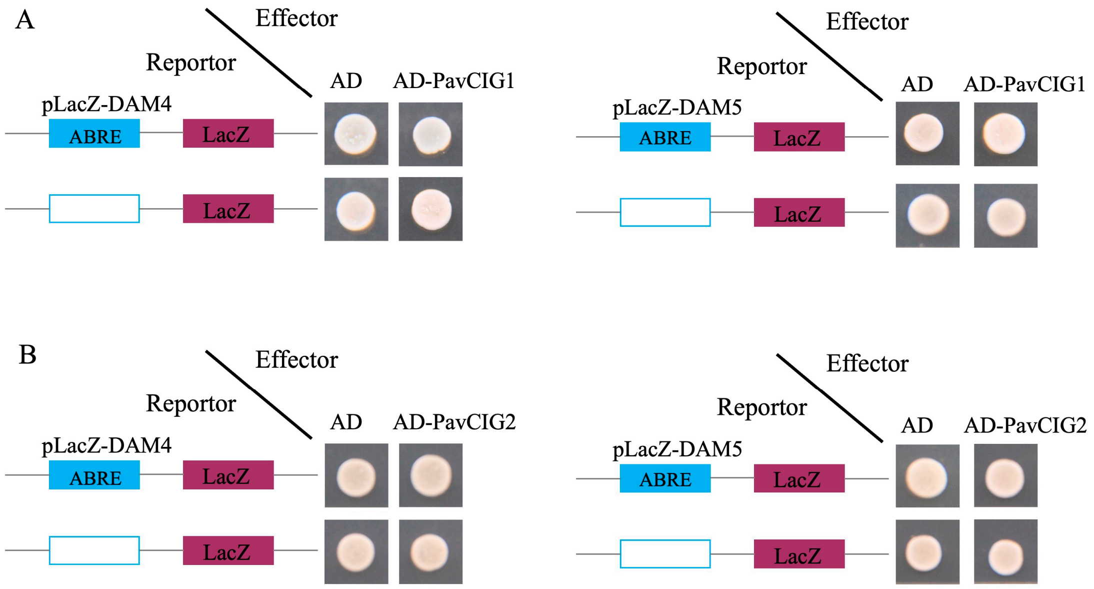

Supplementary Figure S1. Yeast one-hybrid assay was performed to confirm the interactions between PavCIG1/2 and the PavDAM4/5 promoters. (A) Interactions between PavCIG1 and PavDAM4/5. (B) Interactions between PavCIG2 and PavDAM4/5. Empty pLacZ vector was used as the control.

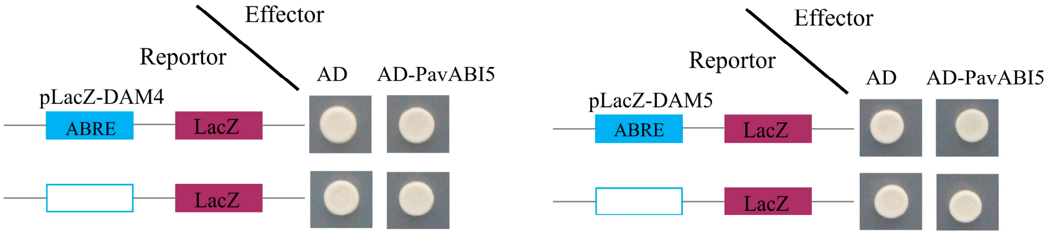

Supplementary Figure S2. Interactions between PavABI5 and PavDAM4/5. Yeast one-hybrid assay was performed to confirm the interactions between PavABI5 and the PavDAM4/5 promoters. Empty pLacZ vector was used as the control.

Supplementary Table S1 List of primer sequences used in this study

Gene	Forward primer (5'-3')	Reverse primer (5'-3')
**For gene cloning**		
PavABI5	AAACATGGGGGTTTCGGAGT	CTCTGTCTTGCATATCATAAGGGG
PavCIG1	AGCTCACTCTCTCATGGATATGATC	CAAAAACATCAATCAGATTGAGAAACT
PavCIG2	AGCTCTACACACACCTCTCTCTC	CCCTACAATCTTTATCAGCGAC
**For real-time qPCR**		

PavACTIN	CGGTATTGCAGACGGATGAGC	GGTACTGAGGGATGCAAGGATGG
PavABI5	AGAGGAGGCAGAGAAGGATGATTA	GCATTGTCCTCTCTTAACTGGTTC
PavCIG1	TGCGCACTTCGGACGTCATA	GGACTTCTTCTTGTTGGGCTGT
PavCIG2	GAAGAATTTTAGCGTGGATATGG	TGTCATCCCAACTCAAGTAGCC
PavDAM1	AGGGGACGATGAAAATGATGAGG	GATAACTGCCACCTCAGATTCACA
PavDAM2	GTTAGAACAACTGGTGGACGCAAG	AGCTGCTGATGGTTGCATTTGTGG
PavDAM3	AGCCAACAACCAGTTAAGGCAGAC	GTCATCTCCAAGAGAGTGAGCACT
PavDAM4	AGCCAACAACCAGTTAAGGCAGAC	CAAGAGAGAGACTGAGAGCACTGTT
PavDAM5	AATTGAATGATCAAGAGATTGAA	CAGCTCTTCCTTAGTTTCCATG
PavDAM6	GAGTGAGATCATGTCACTGGAGAA	AGCTGGTAGAGGTGGCCATTGTG
**For subcellular localization**		
PavABI5	ACCAGTCTCTCTCTCAAGCTTATGGGGGTTTCGGAGTCCGAA	GCTCACCATGGATCCAAGCTTTAAGGGGCAACTATGACACCT
**For promoter cloning**		
Pro-PavCIG1	GACCAACTTTCCCTCCTTGATTA	CTTGCGTCGGATAACGAACTC
Pro-PavCIG2	TGATGCTCCATTTTTCAGGG	TGGTCGGGGGAGTCAGAAA
Pro-PavDAM4	ATGAATACTGAGGTGTCGTGTAACG	TTCTCCCTCATCATTTTCACCAT
Pro-PavDAM5	AAGATGACTGCTCCGACGA	CAGATAACTCTGCAGCTTTC
**For yeast one-hybrid assay**		
pB42AD-PavABI5	TGCCTCTCCCGAATTCATGGGGGTTTCGGAGT	TCCAAAGCTTCTCGAGTCATAAGGGGCAACTA
pB42AD-PavCIG1	TGCCTCTCCCGAATTCATGGATATGATCTTCG	TCCAAAGCTTCTCGAGTCAGATTGAGAAACTC
pB42AD-PavCIG2	TGCCTCTCCCGAATTCATGAACATGTTCTCCG	TCCAAAGCTTCTCGAGTCAGAAAGAGAAACTC
pLacZ-PavCIG1	TATTGGATCGGAATTCACCAACTTTCCCTCCT	GAGCACATGCCTCGAGATATTTGAGTGGTGTT
pLacZ-PavCIG2	TATTGGATCGGAATTCGCTGAGCTCGGAGTGG	GAGCACATGCCTCGAGCACGGACTGTGTGGAG
pLacZ-PavDAM4	TATTGGATCGGAATTCGAATGATTACCTGCAT	GAGCACATGCCTCGAGCAACCACTTTTCAAAT
pLacZ-PavDAM5	TATTGGATCGGAATTCATGACTGCTCCGACGA	GAGCACATGCCTCGAGTTCAAACCAACCACTT
**For Luciferase reporter assays**		
pGreen-proPavCIG1	CTTGATATCGAATTCCTGCAGACCAACTTTCCCTCCTTGATT	GCTCTAGAACTAGTGGATCCTATATTTGAGTGGTGTTTCTG
pGreen-proPavCIG2	CTTGATATCGAATTCCTGCAGAGCTCGGAGTGGGAGCCACGT	GCTCTAGAACTAGTGGATCCACGGACTGTGTGGAGGCGCCA
pRI101-ABI5	ACATATGCCCGTCGACATGGGGGTTTCGGAGT	TACCCCCGGGGTCGACTCATAAGGGGCAACTA
